# 

**Published:** 1857-04

**Authors:** 


*American Dental Convention.*—We take great pleasure in publishing the following notice of the next meeting of the American Dental Convention, and we embrace the occasion to express the hope that it will be as well attended as the last.—Eds.

---

The third annual meeting of the "*American Dental Convention*" will be held in the city of Boston, on Tuesday the 1st of August next, at 12 o'clock, M.

This convention is open to "*all practicing dentists,*" and it is hoped that the profession will be largely represented.

State and local societies are expected to send one or *more* delegates.

At the last annual meeting the following resolution was adopted:

*Resolved,* That the corresponding secretary request all persons having any thing new and useful to the dental profession to present it at the next meeting.

W. W. Allpret,
*Corresponding Secretary.*

*Chicago, March* 16*th,* 1857.

